# Toxicological and Biochemical Analyses Demonstrate the Absence of Lethal or Sublethal Effects of *cry1C-* or *cry2A*-Expressing *Bt* Rice on the Collembolan *Folsomia candida*

**DOI:** 10.3389/fpls.2018.00131

**Published:** 2018-02-06

**Authors:** Yan Yang, Bing Zhang, Xiang Zhou, Jörg Romeis, Yufa Peng, Yunhe Li

**Affiliations:** ^1^State Key Laboratory for Biology of Plant Diseases and Insect Pests, Institute of Plant Protection, Chinese Academy of Agricultural Sciences, Beijing, China; ^2^Research Division Agroecology and Environment, Agroscope, Zurich, Switzerland; ^3^Institute of Tropical Agriculture and Forestry, Hainan University, Haikou, China

**Keywords:** *Bt* rice pollen, *Bt* rice leaf, environmental risk assessment, ELISA, non-target effects, enzyme activity

## Abstract

Assessing the potential effects of insect-resistant genetically engineered (GE) plants on collembolans is important because these common soil arthropods may be exposed to insecticidal proteins produced in GE plants by ingestion of plant residues, crop pollen, or root exudates. Laboratory studies were conducted to evaluate the potential effects of two *Bacillus thuringiensis* (*Bt*)-rice lines expressing Cry1C and Cry2A in pollen and leaves and of their non-*Bt* conventional isolines on the fitness of the collembolan *Folsomia candida* and on the activities of its antioxidant-related enzymes, superoxide dismutase and peroxidase, and of its detoxification-related enzymes, glutathione reductase and glutathione *S*-transferase. Survival, development, reproduction, and the intrinsic rate of increase (*r*_m_) were not significantly reduced when *F. candida* fed on the *Bt* rice pollen or leaf powder than on the non-*Bt* rice materials; these parameters, however, were significantly reduced when *F. candida* fed on non-*Bt* rice pollen or non-*Bt* leaf-based diets containing the protease inhibitor E-64 at 75 μg/g. The activities of the antioxidant-related and detoxification-related enzymes *in F. candida* were not significantly affected when *F. candida* fed on the *Bt* rice materials, but were significantly increased when *F. candida* fed on the non-*Bt* rice materials containing E-64. The results demonstrate that Cry1C and Cry2A are not toxic to *F. candida*, and also indicate the absence of unintended effects on the collembolan caused by any change in plant tissue nutritional composition due to foreign gene transformation.

## Introduction

Multiple genetically engineered (GE) rice lines producing Cry1 and Cry2 proteins derived from *Bacillus thuringiensis* (*Bt* rice) have been developed in China and many of these lines can efficiently control target pests such as *Chilo suppressalis* and *Cnaphalocrocis medinalis* (both Lepidoptera: Crambidae) (Li et al., 2016). Planting of *Bt* rice cultivars thus has great potential for reducing insecticide applications and to thereby benefit the environment and also human and animal health (Li et al., 2016; National Academies of Sciences Engineering Medicine [NASEM], 2016). Nevertheless, the cultivation of GE plants in general and of *Bt* rice, in particular, remains controversial because of safety concerns, one of which is the potential risk to valuable non-target organisms (Romeis et al., 2008; Li et al., 2017). Assessing the potential effects of *Bt* rice on non-target organisms is therefore an important part of the environmental risk assessment that must be conducted before the *Bt* rice can be commercially planted.

Recent studies have investigated the potential effects of *Bt* rice cultivars producing Cry1 and Cry2 proteins on non-target arthropods under laboratory or field conditions (reviewed by Li et al., 2016). Most of these studies have focused on plant-dwelling arthropods including predators belonging to different orders (Tian et al., 2010; Han et al., 2011, 2014, 2017; Li et al., 2014b, 2015; Wang et al., 2015, 2017; Meng et al., 2016), parasitic Hymenoptera (Han et al., 2015; Tian et al., 2017), and the silkworm *Bombyx mori* (Lepidoptera) (Yang et al., 2014). In all cases, these studies found that Cry proteins produced by *Bt* rice plants are very specific to target pest species in the order Lepidoptera and are not toxic to non-target species that do not belong to this order of insects (Li et al., 2016). Among the non-target species tested, only *B. mori* was adversely affected by consuming large amounts of *Bt* rice pollen containing Cry1C and Cry2A proteins (Yang et al., 2014). Sensitivity of *B. mori* to these proteins was expected because this species belongs to the target order Lepidoptera. The negative effects on *B. mori*, however, were only observed at exposure levels that far exceeded those expected under natural conditions, and the researchers, therefore, concluded that the planting of *Bt* rice would pose a negligible risk to *B. mori* (Yang et al., 2014).

Collembolans (springtails) are common arthropods in agricultural soils (Al-Deeb et al., 2003; Bai et al., 2010). In addition to being important consumers of plant residues and soil fungi, collembolans also help create humus (Bitzer et al., 2002). The common soil collembolan *Folsomia candida* (Collembola: Isotomidae) can be easily maintained in the laboratory and has been widely used as a standard test organism for assessing the non-target effects of insecticides and insecticidal GE plants (Fountain and Hopkin, 2005; Romeis et al., 2013; Yang et al., 2015; Zhang et al., 2017). This species has also been used in non-target risk assessment of *Bt* rice including rice lines expressing Cry1Ab or Cry1Ab/1Ac (Bai et al., 2010, 2011; Yuan et al., 2011, 2013, 2014). To our knowledge, information regarding the effects of *Bt* rice lines expressing the *cry1C* or *cry2A* genes on *F. candida* is limited to one study in which the collembolan was fed purified Cry1C and Cry2A proteins at concentrations that were 10 times higher than those in rice tissues. The results showed that *F. candida* was not sensitive to the Cry proteins (Yang et al., 2015). To date, the effects of feeding *F. candida* plant tissues from *Bt* rice lines containing Cry1C or Cry2A proteins (rather than purified proteins) have not been reported.

We therefore assessed the potential effects of ingestion of *Bt* rice pollen and leaves containing Cry1C or Cry2A proteins on *F. candida* with the hypothesis that consumption of these Bt rice material will not significantly affect the fitness of *F. candida.* Because plant tissues were used rather than purified toxins, we expected that the assessment would cover both the direct effects from the Cry proteins as well as possible indirect effects caused by unintended changes in plant composition as a consequence of the genetic transformation.

## Materials and Methods

### Plant Material

The transgenic *Bt* rice cultivars T1C-19 and T2A-1 and their corresponding non-transformed near-isoline Minghui 63 (MH63) were used in the experiments. T1C-19 plants express a modified *cry1C* gene, and T2A-1 plants express a modified *cry2A* gene; the proteins encoded by both genes target lepidopteran rice pests. The non-*Bt* rice line MH63 is an elite indica restorer line for cytoplasmic male sterility in China.

The rice lines were simultaneously planted in three adjacent plots at the experimental field station of the Institute of Plant Protection, CAAS, near Langfang City, Hebei Province, China (39.5°N, 116.4°E). Each plot was approximately 0.1 ha, and the plots were separated by a 1-m ridge. The rice seeds were sown in a seeding bed on May 6, 2015. When the seedlings were at the four-leaf stage, they were transplanted in the field (June 14, 2015). The plants were cultivated according to local agricultural practices but without pesticide sprays.

A previous study showed that the Cry protein concentrations in the two transgenic rice lines are higher in the leaves than in the stems or roots and are highest in the leaves at the seedling stage (Wang et al., 2016). Rice leaves were therefore collected from >50 randomly selected seedlings before they were transplanted in the field on June 10, 2015. The collected leaves were immediately frozen in liquid nitrogen, lyophilized, ground to a fine powder, and stored at -20°C until they were used in the experiments.

When rice plants in the plots reached the flowering stage, rice pollen was collected daily from 3 to 13 September 2015 by shaking the rice tassels in a plastic bag. The collected pollen was air dried at room temperature for 48 h and subsequently passed through a fine mesh (0.125 mm) to remove anthers and contaminants. Pollen collected from each rice line was pooled and stored at -20°C until used in the experiments.

### Test Insects

The *F. candida* specimens used in the current study were obtained from the same permanent laboratory colony as described in our previous studies (Yang et al., 2015; Zhang et al., 2017). The collembolans used in the experiments were 12 days old, which followed the OECD guidelines (Organisation for Economic Cooperation and Development [OECD], 2016) and which ensured that the specimens were mature (Snider, 1973). To obtain 12-day-old insects, we placed *F. candida* adults in Petri dishes with plaster in the bases and allowed the females to oviposit for 48 h before all adults were removed. The eggs hatched after approximately 7 days and the neonates that hatched on a single day were subsequently fed on the baker’s yeast for 11 days and were then used in the experiments.

### Rice Pollen Experiment

Our preliminary experiments showed that *F. candida* survival and development were similar on rice pollen (MH63) and on baker’s yeast, which is a diet that is favored by the collembolan (*unpublished data*). This indicated that rice pollen is a suitable food for *F. candida* and can be used in dietary exposure experiments.

For the experiment, 12-day-old *F. candida* were randomly selected, individually placed in Petri dishes (diameter 35 mm; height 10 mm; with plaster in the base), and subjected to one of the following dietary treatments: (i) MH63 rice pollen (non-*Bt* rice pollen; negative control); (ii) T1C-19 rice pollen (*Bt* pollen containing Cry1C); (iii) T2A-1 rice pollen (*Bt* pollen containing Cry2A); and (iv) MH63 rice pollen mixed with E-64 [*trans*-epoxysuccinyl-L-leucylamido (4-guanidino) butane], which served as a positive control. The protease inhibitor E-64 was purchased from Sigma–Aldrich (St. Louis, MO, United States) and was used as a positive control because it is known to be toxic to *F. candida* (Yang et al., 2015). For preparation of the positive control, stock solutions of E-64 were diluted with distilled water to a defined concentration and then mixed with non-*Bt* rice pollen (75 μg/g pollen dry weight [DW]). To ensure that the control and pollen treatments were prepared similarly, the same volume of distilled water was mixed with *Bt* and non-*Bt* rice pollen. All of the prepared pollen diets were lyophilized and ground into powder 3 days before the initiation of the experiment and were stored at -20°C until used. Each treatment was represented by 50 replicates (one collembolan and dish per replicate). The diets were renewed every 2 days to prevent the degradation of the test compounds. Survival, the number of fecal pellets produced, and the numbers of eggs and offspring produced by each collembolan were recorded twice daily (9:00 a.m. and 9:00 p.m.). Every seven days, the surviving individuals were photographed with a photo-microscope, and body length and head width were measured using a scale in the microscope. The experiment, which was conducted in a climate chamber at 20 ± 1°C with 70 ± 5% RH and a 12-h light/12-h dark cycle, was terminated after 35 days.

To estimate the intrinsic rate of natural increase (*r*_m_) of *F. candida*, individuals in each treatment were randomly assigned to one of three groups (16 or 17 individuals per group), resulting in three replicate groups per treatment. With the observed data, the *r*_m_ was calculated per group using the equation described in our previous study (Zhang et al., 2017).

### Rice Leaf Experiment

Powder from leaves alone is known to be an unsuitable food for *F. candida* (Yu et al., 1997; Romeis et al., 2003). Our preliminary experiments revealed that rice leaf powder mixed with baker’s yeast at a ratio of 10:1 (w: w) can support the survival, development, and reproduction of *F. candida* (*unpublished data*), and this mixture was used as the leaf-based diet in the current study.

The method used for the leaf-feeding experiment was similar to that used for the pollen-feeding experiment. The 12-day-old *F. candida* larvae were fed the following diets: (i) MH63 leaf powder mixed with baker’s yeast (leaf-based diet; negative control); (ii) T1C-19 leaf-based diet; (iii) T2A-1 leaf-based diet; and (iv) MH63 leaf-based diet containing E-64. Each treatment was represented by 50 replicates (one individual and dish per replicate), and the same life table parameters including the *r*_m_ were recorded as described for the pollen-feeding experiment.

### Uptake of Cry Protein by *F. candida* during the Feeding Experiments

To estimate the uptake of Cry1C or Cry2A protein by *F. candida* that fed on diets containing *Bt* rice pollen or leaf powder as described above, a separate assay was performed in which >30 Petri dishes (diameter 90 mm; height 10 mm), each containing >100 *F. candida* (12 days old) were provided with the *Bt* rice diets (pollen or leaf-based) or corresponding non-*Bt* diets as described above. After 7, 21, and 35 days of feeding, four samples per treatment (with 50–60 individuals per sample) were collected from different Petri dishes, resulting in a total of 72 samples (36 samples for pollen and 36 for leaves). The samples were frozen at -60°C for later ELISA analysis according to the methods described in our previous study (Li et al., 2015).

### Stability and Bioactivity of Cry Proteins in the Diets

To evaluate the stability and bioactivity of Cry proteins in pollen or leaf-based diets during the feeding experiments, three 2- to 3-mg (FW) subsamples were collected from fresh diets that had been kept at -20° and from diets that had been exposed to *F. candida* for 2 days. The concentration and bioactivity of Cry proteins in the diets were analyzed by ELISA and sensitive-insect bioassays, respectively, according to the methods described in our previous study (Li et al., 2015).

### Determination of Enzyme Activity

*Folsomia candida* (12 days old) were placed in Petri dishes (diameter 90 mm; height 10 mm; between 50 and 60 specimens per dish; >50 dishes in total) and exposed to non-*Bt* or *Bt* rice pollen or leaf-based diet or non-*Bt* rice pollen or leaf-based diet containing E-64 for 0, 7, 14, or 21 days as described before. At each sampling date, *F. candida* samples (200–300 individuals per sample, one sample per diet) were collected and stored at -20°C before the activities of the following enzymes were quantified in each sample: the antioxidant-related enzymes superoxide dismutase (SOD) and peroxidase (POD), and the detoxification-related enzymes glutathione (GR) and glutathione *S*-transferase (GST). The activities of these enzymes have been widely used as indicators of adverse effects caused by stomach poisons in *F. candida* and other arthropods (Bai et al., 2011; Yuan et al., 2011; Yang et al., 2015; Zhang et al., 2017). All enzyme activities were measured with enzyme kits from Nanjing Jiancheng Ltd., Co. (Nanjing, China) as described in our previous study (Zhang et al., 2017).

### Data Analysis

Dunnett’s tests were used to analyze the difference between the treatments and the negative control for the following parameters: body length, head width, number of fecal pellets, number of eggs, and the intrinsic rate of increase. Hatching rates were analyzed by one-way ANOVA followed by HSD tests. Survival rates were analyzed with the Kaplan–Meier procedure and Logrank test. Cry protein concentrations and enzyme activities in *F. candida* collected on different days during the feeding assay were analyzed by repeated measures (RM-) ANOVA. In addition, Student’s *t*-tests were used to compare Cry protein concentrations in the fresh pollen/leaf diets vs. pollen/leaf diets exposed to *F. candida* for 2 days. Chi-square tests were used to compare the mortalities of the *C. suppressalis* larvae in the sensitive-insect bioassay. All statistical analyses were conducted using the software package SPSS (version 15.0 for Windows, 2006). Unless noted otherwise, values are presented as means ± SE.

## Results

### No Effects on Fitness of *F. candida* by Feeding on *Bt* Rice Pollen

**FIGURE 1:**
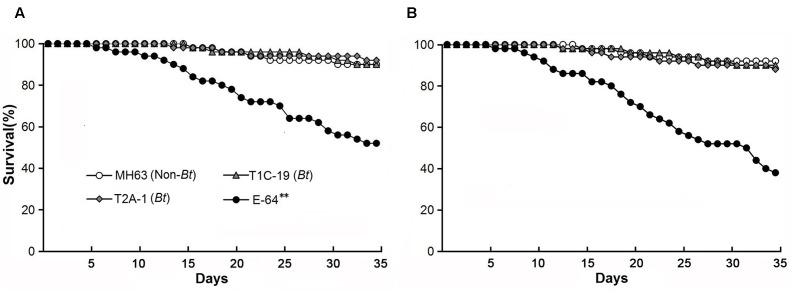
Survival of *Folsomia candida* on a diet consisting of rice pollen **(A)** or a mixture of rice leaf powder and baker’s yeast (10:1) **(B)**. The pollen and leaf power were obtained from either of two *Bt* rice lines (T1C-19: Cry1C, and T2A-1: Cry2A) or their non-*Bt* near isoline MH63 (negative control). For the positive control, the corresponding non-*Bt* rice tissue was supplemented with E-64. Asterisks indicate a significant difference between the treatment and the negative control (Logrank test, *P* < 0.01) (*n* = 50).

**FIGURE 2:**
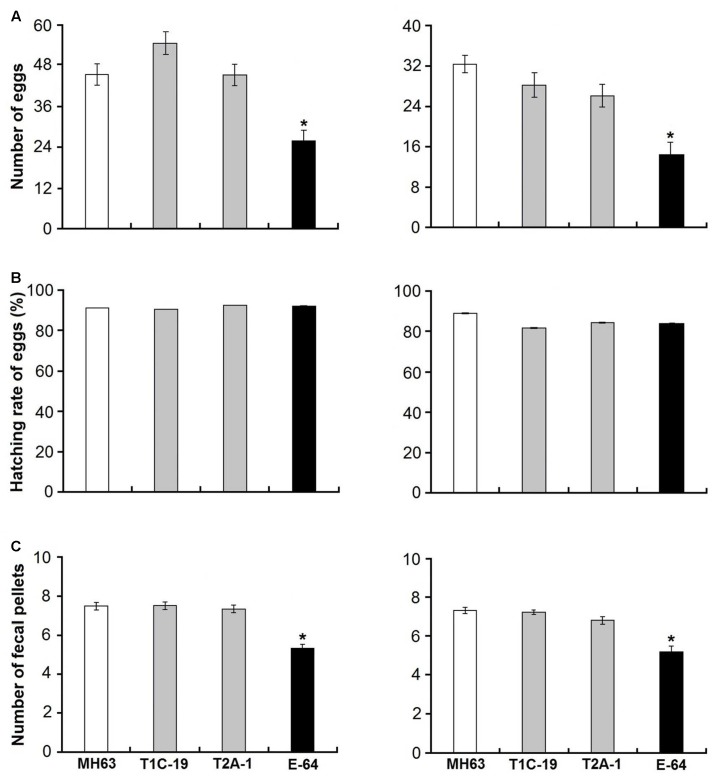
Fecundity (numbers of eggs) produced per individual **(A)**, hatching rate of eggs **(B)**, and numbers of fecal pellets produced per individual **(C)** of *F. candida* fed on a diet consisting of rice pollen (left) or a mixture of rice leaf powder and baker’s yeast (10:1) (right). The rice pollen and rice leaf powder were obtained from either of two *Bt* rice lines (T1C-19: Cry1C and T2A-1: Cry2A) or their non-*Bt* near isoline MH63 (negative control). For the positive control, the corresponding non-*Bt* rice tissue was supplemented with E-64 (positive control) for 35 days. Values are means ± SE, *n* = 50. Asterisks indicate a significant difference between the treatment and the negative control (MH63) (Dunnett’s test, *P* < 0.05).

The survival rates were >90% when *F. candida* fed on either *Bt* rice pollen (T1C-19 or T2A-1) or non-*Bt* rice pollen for 35 days, and there was no significant difference between any *Bt* pollen treatment and the control pollen treatment (χ^2^< 0.01, *P* = 0.99 for T1C-19; χ^2^ = 0.12, *P* = 0.73 for T2A-1) (**Figure 1A**). However, the survival rate was significantly reduced when *F. candida* fed on non-*Bt* pollen containing E-64 (χ^2^ = 17.660, *P* < 0.001). Similarly, the mean body length and head width of *F. candida* were not affected by ingestion of *Bt* rice pollen (*P* > 0.10 for all sampling dates) (**Table 1**). In addition, the number of eggs produced per individual and the number of fecal pellets produced per individual were not affected by feeding on *Bt* rice pollen (**Figure 2**) (Dunnett’s tests; T1C-19 pollen: *P* = 0.27 for number of eggs, and *P* = 1.00 for a number of fecal pellets; T2A-1 pollen: *P* = 1.00 for number of eggs, and *P* = 0.93 for a number of fecal pellets). All of these parameters, however, were significantly reduced when *F. candida* fed on the non-*Bt* rice pollen containing E-64 (Dunnett’s tests, all *P* < 0.01). Interestingly, the egg hatching rate of *F. candida* did not significantly differ among the diets (one-way ANOVA; *F*_3,128_ = 1.11, *P* = 0.35).

**Table 1:** Body length and head width of *Folsomia candida* that fed on a diet consisting of non-*Bt* pollen/leaf (MH63, negative control), *Bt* pollen/leaf (T1C-19: Cry1C, and T2A-1: Cry2A), or non-*Bt* pollen/leaf plus E-64 protein (E-64, positive control) for 35 days.

		Treatment							
Parameters	Day	Pollen diet				Leaf diet			
		MH63	T1C-19	T2A-1	E-64	MH63	T1C-19	T2A-1	E-64
Body length (mm)	7	1.071 ± 0.009	1.067 ± 0.010	1.077 ± 0.009	1.047 ± 0.009^∗^	1.111 ± 0.010	1.081 ± 0.012	1.083 ± 0.012	1.058 ± 0.013^∗^
	14	1.214 ± 0.010	1.205 ± 0.013	1.226 ± 0.012	1.157 ± 0.014^∗^	1.194 ± 0.010	1.197 ± 0.012	1.188 ± 0.012	1.122 ± 0.015^∗^
	21	1.287 ± 0.011	1.288 ± 0.014	1.287 ± 0.013	1.191 ± 0.013^∗^	1.252 ± 0.009	1.255 ± 0.010	1.278 ± 0.011	1.161 ± 0.015^∗^
	28	1.346 ± 0.012	1.347 ± 0.014	1.346 ± 0.014	1.233 ± 0.017^∗^	1.313 ± 0.009	1.223 ± 0.010	1.348 ± 0.011	1.166 ± 0.029^∗^
	35	1.399 ± 0.014	1.408 ± 0.015	1.407 ± 0.016	1.292 ± 0.008^∗^	1.385 ± 0.008	1.381 ± 0.010	1.406 ± 0.080	1.216 ± 0.019^∗^
Head width (mm)	7	0.204 ± 0.002	0.204 ± 0.001	0.204 ± 0.001	0.203 ± 0.001^∗^	0.218 ± 0.002	0.213 ± 0.002	0.213 ± 0.002	0.205 ± 0.021^∗^
	14	0.226 ± 0.001	0.225 ± 0.002	0.228 ± 0.001	0.219 ± 0.001^∗^	0.235 ± 0.002	0.235 ± 0.002	0.235 ± 0.002	0.219 ± 0.024^∗^
	21	0.238 ± 0.001	0.235 ± 0.002	0.236 ± 0.001	0.224 ± 0.001^∗^	0.242 ± 0.002	0.239 ± 0.003	0.243 ± 0.002	0.225 ± 0.023^∗^
	28	0.246 ± 0.001	0.244 ± 0.002	0.243 ± 0.001	0.228 ± 0.002^∗^	0.248 ± 0.002	0.250 ± 0.001	0.251 ± 0.002	0.231 ± 0.034^∗^
	35	0.253 ± 0.001	0.252 ± 0.002	0.251 ± 0.002	0.235 ± 0.002^∗^	0.257 ± 0.001	0.258 ± 0.001	0.259 ± 0.002	0.236 ± 0.032^∗^

*Rice leaf powder was mixed with baker’s yeast (10:1). The feeding experiment began with 12-day-old, mature insects; in the table, Day refers to days after the experiment began. Values are means ± SE, n = 50. Means in a row followed by an asterisk differ significantly from the respective negative control, i.e., MH63 pollen or leaf-based diets (P > 0.05, Dunnett’s test).*

The *r*_m_ values were 0.14 ± 0.001, 0.14 ± 0.003, 0.14 ± 0.005, and 0.11 ± 0.003 when *F. candida* fed on the pollen from non-*Bt* plants, T1C-19, T2A-1, and non-*Bt* plants containing E-64, respectively. The mean *r*_m_ values did not significantly differ between *Bt* and non-*Bt* pollen treatments (Dunnett’s tests; *P* = 0.66 for T1C-19 pollen, *P* = 0.99 for T2A-1 pollen), but the mean *r*_m_ value was significantly reduced for *F. candida* that fed on the non-*Bt* pollen containing E-64 (*P* = 0.004).

### Concentrations of Cry Proteins in *F. candida* Feeding on *Bt* Rice Pollen

As indicated by ELISA measurements, *F. candida* that fed on the *Bt* rice pollen diets contained Cry proteins. The concentration of Cry1C detected in *F. candida* on days 7, 21, and 35 was 38.49 ± 0.23, 41.89 ± 0.76, and 42.54 ± 1.44 ng/g DW, respectively, and the concentration significantly increased over time (RM-ANOVA, *F*_1,3_ = 2963.9, *P* < 0.001). The concentration of Cry2A detected in *F. candida* on days 7, 21, and 35 was 115.12 ± 6.17, 125.10 ± 0.19, and 252.85 ± 1.74 ng/g DW, respectively, and the concentration significantly increased over time (*F*_1,3_ = 79431.2, *P* < 0.001). No Cry protein was detected in *F. candida* that fed on the non-*Bt* rice pollen.

### Stability and Bioactivity of Cry Proteins in Pollen Diets

According to ELISA measurements, the concentration of Cry1C protein in the freshly prepared Cry1C rice pollen diet was 1.82 ± 0.02 μg/g of pollen, and the concentration significantly decreased to 1.16 ± 0.02 μg/g of pollen after a 2-day feeding exposure to *F. candida* (Student’s *t*-test; *t* = 29.7, df = 6, *P* < 0.001). The concentration of Cry2A detected in the freshly prepared Cry2A rice pollen diet was 26.41 ± 0.19 μg/g of pollen, and the concentration significantly decreased to 24.07 ± 0.12 μg/g of pollen after a 2-day feeding exposure to *F. candida* (Student’s *t*-test; *t* = 10.4, df = 6, *P* < 0.001). No *Bt* protein was detected in non-*Bt* rice pollen.

The sensitive-insect bioassay showed that the mortality of *C. suppressalis* larvae was 3.3 ± 3.3% when the larvae were fed a diet containing the extract from non-*Bt* rice pollen for 7 days. The mortalities were 86.7 ± 3.3% or 83.3 ± 3.3% when *C. suppressalis* larvae were fed diets containing the extract from fresh Cry1C pollen (T1C-19) or Cry1C pollen that had been exposed to *F. candida* for 2 days. The mortalities were 80.0 ± 5.8% or 76.7 ± 3.3% when *C. suppressalis* larvae were fed diets containing the extract from fresh Cry2A pollen (T2A-1) or Cry2A pollen that had been exposed to *F. candida* for 2 days. Mortalities were not significantly different for larvae that fed on fresh diet vs. 2-day-old diet (Chi-square test; *U* = 0.13, df = 1, *P* = 0.72 for T1C-19 pollen; *U* = 0.10, df = 1, *P* = 0.75 for T2A-1 pollen).

### No Effects on Fitness of *F. candida* by Feeding on *Bt* Rice Leaf Tissue

The survival rates were ≥88% when *F. candida* fed on the *Bt* rice (T1C-19 or T2A-1) leaf-based diets for 35 days, and there was no significant difference between any *Bt* leaf diet treatment and the control treatment (χ^2^ = 0.11, *P* = 0.74 for T1C-19; χ^2^ = 0.53, *P* = 0.47 for T2A-1) (**Figure 1B**). However, the survival rate was significantly reduced when *F. candida* fed on the non-*Bt* leaf-based diet containing E-64 (χ^2^ = 32.17, *P* < 0.001). Similarly, the mean body length and head width were not affected by ingestion of the *Bt* leaf-based diet (*P* > 0.10 for all sampling dates) (**Table 1**). In addition, the number of eggs produced per individual and the number of fecal pellets produced per individual was not affected by feeding on a *Bt* leaf-based diet (**Figure 2**) (Dunnett’s tests; T1C-19 leaves: *P* = 0.37 for number of eggs, and *P* = 0.98 for a number of fecal pellets; T2A-1 leaves: *P* = 0.093 for number of eggs, and *P* = 0.092 for a number of fecal pellets). All of these parameters, however, were significantly reduced when *F. candida* fed on a non-*Bt* leaf-based diet containing E-64 (Dunnett’s tests, all *P* < 0.01). As was the case with the pollen diets, the egg hatching rates of *F. candida* did not significantly differ among leaf-based diets (one-way ANOVA, *F*_3,147_ = 1.73, *P* = 0.16).

The *r*_m_ values of *F. candida* were 0.138 ± 0.004, 0.138 ± 0.001, 0.136 ± 0.003, and 0.117 ± 0.007 for *F. candida* that fed on a leaf-based diet from non-*Bt* plants, T1C-19 plants, T2A-1 plants, and non-*Bt* plants containing E-64, respectively. The mean *r*_m_ values did not significantly differ between *Bt* and non-*Bt* diet treatments (Dunnett’s tests, *P* = 1.0 for T1C-19 leaves, *P* = 0.969 for T2A-1 leaves), except that the mean *r*_m_ value was significantly reduced when *F. candida* fed on the non-*Bt* diet containing E-64 (*P* = 0.017).

### Concentrations of Cry Proteins by *F. candida* Feeding on *Bt* Rice Leaf Tissue

As indicated by ELISA measurements, *F. candida* that fed on a *Bt* rice leaf-based diet contained Cry proteins. The concentration of Cry1C detected in *F. candida* on days 7, 21, and 35 was 39.19 ± 0.24, 41.99 ± 1.09, and 64.60 ± 5.83 ng/g DW, respectively, when the collembolan fed on a T1C-19 leaf-based diet, and the concentration significantly increased over time (RM-ANOVA, *F* = 458.1, df = 3, *P* < 0.001). The concentration of Cry2A detected in *F. candida* on days 7, 21, and 35 was 147.53 ± 5.33, 140.08 ± 5.84, and 196.53 ± 0.19 ng/g DW, respectively, when the collembolan fed on a T2A-1 leaf-based diet, and the concentration significantly increased over time (*F*_1,3_ = 7628957.0, *P* < 0.001). No Cry protein was detected in *F. candida* that fed on a leaf-based diet from non-*Bt* rice plants.

### Stability and Bioactivity of Cry Proteins in the Leaf-Based Diets

According to ELISA measurements, the original concentrations of Cry1C and Cry2A in the *Bt* rice leaf-based diets were 1.55 ± 0.03 and 16.38 ± 0.28 μg/g DW diet, respectively. After a 2-day feeding exposure, the contents had significantly decreased to 1.44 ± 0.01 and 13.64 ± 0.26 μg/g diet for Cry1C and Cry2A, respectively (Student’s *t*-test; *t* = 4.1, df = 6, *P* = 0.006 for Cry1C, and *t* = 7.1, df = 6, *P* < 0.001 for Cry2A). No Cry protein was detected in the leaf-based diet made from non-*Bt* rice plant.

The sensitive-insect bioassay showed that the mortality of *C. suppressalis* larvae was 6.7 ± 3.3% when the larvae fed on a diet containing the extract from the non-*Bt* leaf-based diet for 7 days. The mortality was 90.0 ± 5.8% or 73.3 ± 3.3% when *C. suppressalis* larvae fed on diets containing the extract from a fresh Cry1C leaf-based diet (T1C-19) or from a Cry1C leaf-based diet that had been exposed to *F. candida* for 2 days. The mortality was 90.0 ± 5.8% or 80.0 ± 5.8% when *C. suppressalis* larvae were fed diets containing the extract from the fresh Cry2A leaf-based diet (T2A-1) or a Cry2A leaf-based diet that had been exposed to *F. candida* for 2 days. Mortalities were not significantly different for larvae that fed on fresh vs. 2-day-old diet (Chi-square test; *U* = 2.78, df = 1, *P* = 0.10 for Cry1C diet; *U* = 1.18, df = 1, *P* = 0.28 for Cry2A diet).

### No Effects on Enzyme Activities in *F. candida* by Feeding *Bt* Rice Pollen or Leaf Tissue

**FIGURE 3:**
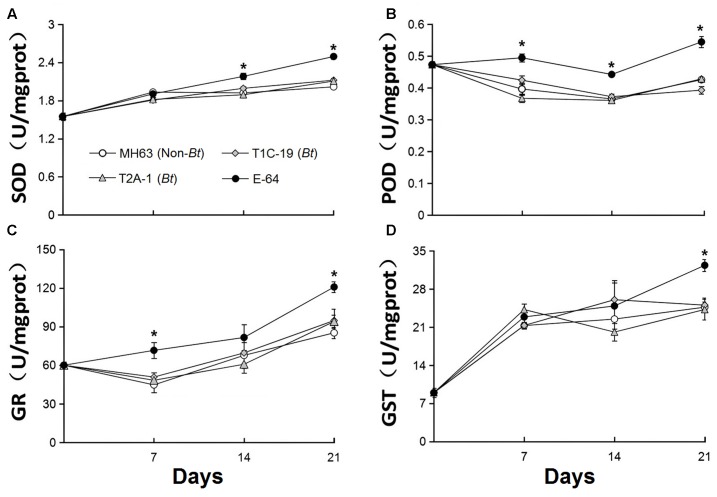
Enzyme activities (**A**: peroxidase, POD; **B**: superoxide dismutase, SOD; **C**: glutathione, GR; and **D**: glutathione *S*-transferase, GST) in *F. candida* that fed on a diet consisting of non-*Bt* rice pollen (MH63, negative control), *Bt* rice pollen containing Cry1C or Cry2A (T1C-19 or T2A-1), or non-*Bt* rice pollen containing E-64 protein (positive control) for 35 days. Values are means ± SE, *n* = 4. Asterisks indicate a significant difference between the treatment and the negative control (MH63) (*P* < 0.05).

**FIGURE 4:**
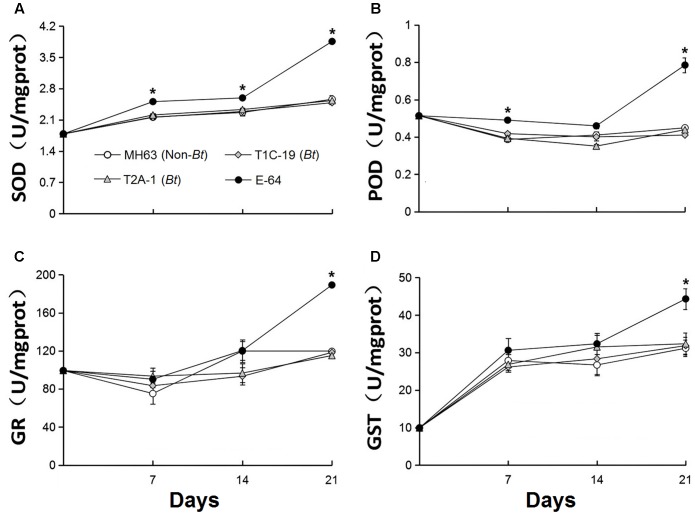
Enzyme activities (**A**: POD, **B**: SOD, **C**: GR, and **D**: GST) in *F. candida* that fed on non-*Bt* rice leaves (MH63, negative control), *Bt* rice leaves containing Cry1C or Cry2A (T1C-19 or T2A-1), or non-*Bt* leaves containing E-64 protein (E-64, positive control) for 35 days. Rice leaf powder was mixed with baker’s yeast (10:1). Values are means ± SE, *n* = 4. Asterisks indicate a significant difference between the treatment and the negative control (MH63) (*P* < 0.05).

The activity of the four enzymes did not significantly differ in *F. candida* that fed on diets containing *Bt* pollen vs. non-*Bt* pollen (**Figure 3**) or *Bt* leaf powder vs. non-*Bt* leaf powder (**Figure 4**) (RM-ANOVA, all *P* > 0.05). In contrast, the activities of the four enzymes were significantly higher (*P* ≤ 0.003) in *F. candida* that fed on the non-*Bt* rice pollen containing E-64 rather than on non-*Bt* rice pollen without E-64 on all test days for POD, on days 14 and 21 for SOD, on days 7 and 21 for GR, and on day 21 for GST (**Figure 3**). The enzyme activities were also significantly higher (*P* ≤ 0.009) in *F. candida* that fed on the non-*Bt* rice leaf-based diet containing E-64 rather than on the same diet without E-64 on all test days for SOD, on days 7 and 21 for POD, and on day 21 for GR and GST (**Figure 4**).

## Discussion

Pollen grains contain multiple organic and inorganic nutrients, such as sugars, starch, amino acids, proteins, lipids, vitamins, and minerals, and can serve as a food source for many arthropods (Li et al., 2010). Plant pollen has therefore been commonly used in dietary exposure assays with bees, lacewings, and ladybird beetles; such assays are essential components of non-target risk assessment of insect-resistant GE crops (Li et al., 2008, 2015; Wang et al., 2012, 2017; Meissle et al., 2014; Zhang et al., 2014). The current study shows that *F. candida* can survive, develop, and reproduce using rice pollen as a sole food source. The results are consistent with our previous study, in which *F. candida* survived well on only maize pollen (Zhang et al., 2017). In contrast, previous studies found that potato, cotton, and wheat leaves alone are not suitable foods for *F. candida* (Yu et al., 1997; Romeis et al., 2003; Bakonyi et al., 2006). Similarly, we found that the fitness of *F. candida* was significantly reduced when the collembolan fed on rice leaf powder alone (Yang et al., unpublished data). Based on our preliminary results, we developed a rice leaf-based diet in which baker’s yeast was mixed with lyophilized leaf powder at a ratio of 1:10. The results indicate that when fed this diet formula, *F. candida* survival rates ≥88%, which meets the standard for such dietary exposure assays (Romeis et al., 2011).

Dietary exposure assays require an appropriate positive control to confirm that the assay is sensitive, i.e., to confirm that the assay can detect the toxic effects of a test compound (Li et al., 2014a). In the current study, E-64 was used as a positive control because it is readily accepted by *F. candida* and is known to be toxic to the collembolan (Zhang et al., 2017). Our feeding experiments showed that ingestion of *Bt* rice pollen or leaf powder from T1C-19 or T2A-1 rice plants did not reduce the survival, development, reproduction, or the intrinsic rate of natural increase (*r*_m_) of *F. candida*. We found, however, that all of these life table parameters, except for the egg hatching rate, were significantly reduced by the consumption of the pollen or leaf-based diet containing E-64. This result demonstrates that the dietary exposure assays developed in our study were able to detect negative effects, and that they are therefore valid for assessing the effects of *Bt* rice pollen or leaf powder on *F. candida*. The results from the feeding bioassays thus indicate that consumption of *Bt* rice pollen or leaf powder has no adverse effects on *F. candida* individuals or populations. That E-64 did not reduce the egg hatching rate of *F. candida* that fed on the compound indicates that egg hatching is not a sensitive life-table parameter for assessing chemical toxicity to *F. candida*.

In addition to the life-table parameters mentioned above, the activities of two antioxidant-related enzymes, SOD and POD, and two detoxification-related enzymes, GR and GST, were measured, because they are known to be involved in the detoxification of reactive oxygen species (ROS) (Felton and Summers, 1995; Hayes and Strange, 2000). ROS might be induced when insects ingest toxic substances, and high levels of ROS may seriously damage the insects (Felton and Summers, 1995). It follows that an increase in the activity of these enzymes in insects may represent a response to the ingestion of a toxic, ROS-inducing compound. For these reasons, the activities of SOD, POD, GR, and GST have been widely used as indicators of the toxicity of *Bt* proteins and other insecticidal compounds (Bai et al., 2011; Yuan et al., 2011; Yang et al., 2015; Zhang et al., 2017). In the current study, the activities of SOD, POD, GR, and GST in *F. candida* were not affected by feeding on *Bt* rice pollen or leaf-based diets containing Cry1C or Cry2A protein. These results are consistent with previous studies. For example, the activities of SOD and POD in *F. candida* were not affected when the collembolan fed on the yeast mixed with Cry1Ab and Cry1Ac proteins (Yuan et al., 2011). Bai et al. (2011) found that SOD activity was not significantly altered in *F. candida* after ingestion of Cry1Ab-containing rice tissue for 35 days (Bai et al., 2011). Yang et al. (2015) showed that ingestion of pure Cry1C or Cry2A protein did not affect the activities of six enzymes in *F. candida* including antioxidant enzymes (SOD and POD), detoxification enzymes (GR and CES), and the proteases (T-Pro and TPS). More recently, Zhang et al. (2017) reported that SOD and POD were not influenced in *F. candida* that fed on the *Bt* corn pollen containing Cry1Ab/2Aj protein. In both Yang et al. (2015) and Zhang et al. (2017), the activities of these enzymes were significantly increased when *F. candida* ingested diets containing E-64, indicating that the assays used were able to detect toxic dietary effects. The lack of effects of consumption of *Bt* rice materials on life-table parameters further indicates that *F. candida* is not affected by Cry1C and Cry2A.

To quantify the exposure of *F. candida* to Cry protein in the feeding experiments, we measured the stability of the Cry proteins in the diets and the uptake of the proteins by *F. candida*. The results showed that the concentrations of Cry1C and Cry2A proteins in both pollen and leaf-based diets declined significantly during the feeding period, but that >60% of the Cry proteins was still detectable after a 2-day feeding exposure. The ingestion of Cry2A and Cry1C proteins by *F. candida* in the experiments was also confirmed by ELISA. In general, the contents of Cry proteins in *F. candida* increased over time with continually feeding on *Bt* pollen or leaf-based diets, which may be due to increased food consumption with *F. candida* growth. Furthermore, the bioactivity of the Cry proteins in the pollen or leaf-based diets was confirmed in a bioassay with *Bt* protein-sensitive *C. suppressalis* larvae. These results demonstrate that *F. candida* larvae ingested bioactive Cry1C and Cry2A protein in our feeding experiments. Given that collembolans are soil organisms with a broad range of food (Ponge, 1991), the *F. candida* in our study, which were exclusively fed *Bt* rice material, were exposed to Cry proteins at levels much higher than would occur under field conditions. That no lethal or sublethal effects were detected under our worst-case exposure conditions demonstrates that *F. candida* is not sensitive to Cry1C or Cry2A proteins in *Bt* rice pollen and leaves. Our results also provide evidence that the genetic engineering of the rice plants has not resulted in any unintended or unexpected changes in rice that affect *F. candida* (Gong and Wang, 2013; Ladics et al., 2015; Schnell et al., 2015; Devos et al., 2016).

As a surrogate collembolan species, *F. candida* has been commonly used in non-target risk assessment of insecticidal GE plants including cotton, potato, wheat, maize, and rice (Yu et al., 1997; Romeis et al., 2003; Bitzer et al., 2005; Clark and Coats, 2006; Bai et al., 2011; Bakonyi et al., 2011; Yuan et al., 2011, 2013; Zhang et al., 2017). Most studies have reported that ingestion of *Bt* proteins or *Bt* protein-containing plant tissues did not have any adverse effects. Two exceptions are the studies by Bakonyi et al. (2006, 2011), in which *F. candida* produced significantly fewer fecal pellets after consuming powder from *Bt* (Cry1Ab) maize leaves rather than powder from non-*Bt* leaves. The reasons for this effect, however, were not elucidated. In summary, the available data with *F. candida* suggest that the currently used *Bt* Cry1, Cry2, and Cry3 proteins are not toxic to collembolans.

To our knowledge, the current report is the first to assess the potential effects of *Bt* rice pollen or leaves containing Cry1C or Cry2A proteins on *F. candida.* The results from our toxicological and biochemical experiments confirmed that Cry1C and Cry2A are not toxic to *F. candida*. The results also indicated the absence of unintended effects on the collembolan caused by any change in plant tissue nutritional composition due to foreign gene transformation. We therefore conclude that the planting of the *Bt* rice lines will pose a negligible risk to *F. candida*.

## Author Contributions

YL designed the study. BZ and YY performed all of the experiments. YY, YL, BZ, JR, and YP analyzed the data and wrote the manuscript. XZ and YP provided the experimental materials. All authors have read and approved the manuscript for publication.

## Conflict of Interest Statement

The authors declare that the research was conducted in the absence of any commercial or financial relationships that could be construed as a potential conflict of interest.
